# Supplementary figures and images for: Paleolithic occupation of arid Central Asia in the Middle Pleistocene
Source: PLoS One. 2022 Oct 21;17(10):e0273984. doi: 10.1371/journal.pone.0273984 (PMC9586385; doi:10.1371/journal.pone.0273984)

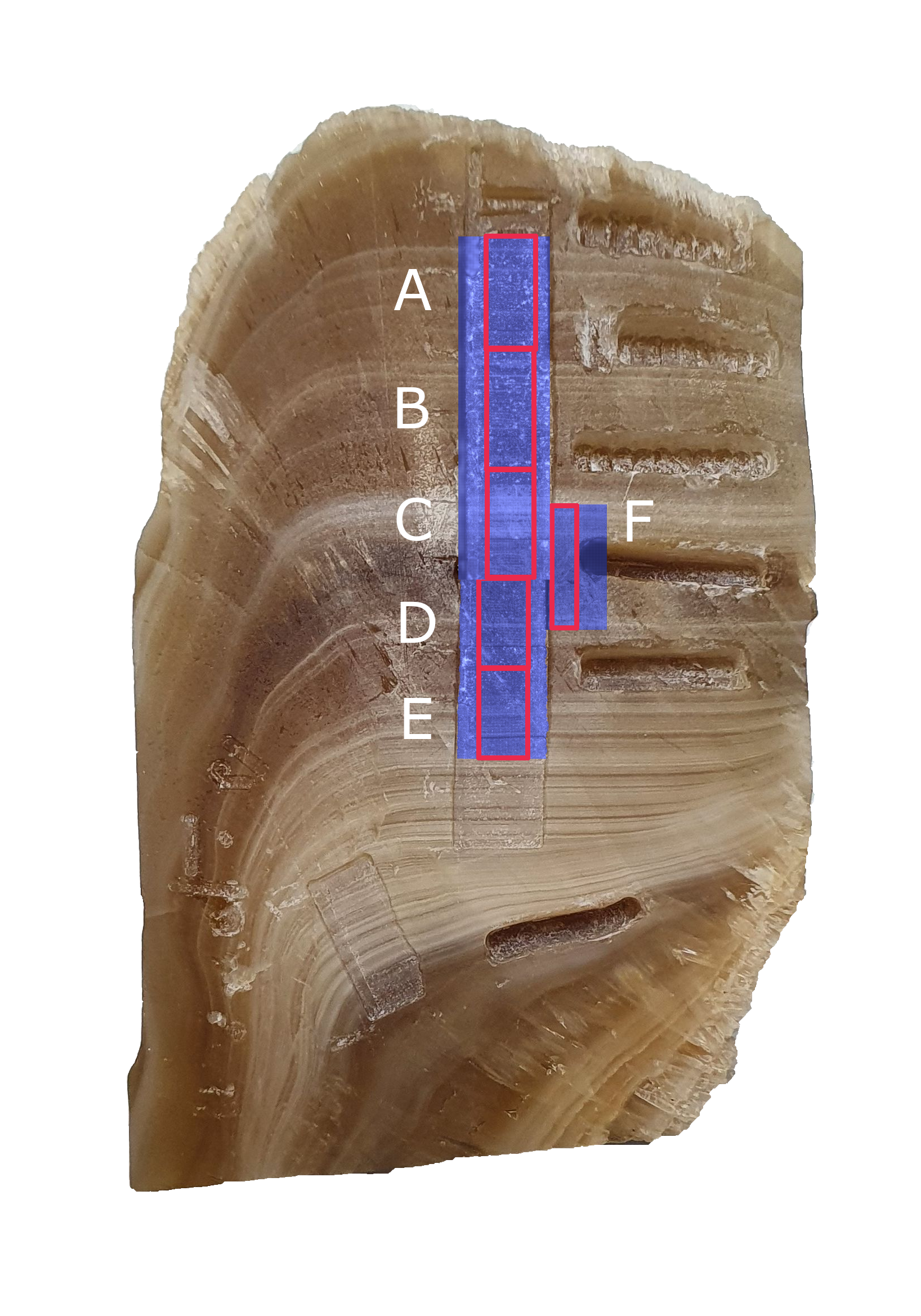

Supplement: S1 Fig — Overview image of stalagmite S-12-4 with the areas measured by CRM (A-F). Blue areas are stitched microscope images obtained with the 20x lens of the CRM and superimposed onto the sample scan. The red rectangles mark areas imaged by CRM. The instrumental settings are identical for all 6 areas and the dimensions are as follows: Area A has a size of 3500 μm x 8000 μm (70000 spectra, distance between measurements 20 μm, integration time 0.1 seconds/measurement). Area B has a size of 3500 μm x 8500 μm (74375 spectra, resolution 20 μm), Area C 3500 x 8200 (71750 spectra, resolution 20 μm), D 3500 μm x 6800 μm (238000 spectra, resolution 10 μm), E 3500 μm x 6800 μm (238000 spectra, resolution 10 μm), and F 1700 μm x 8500 μm (144500 spectra, resolution 10 μm, 0.2 seconds integration time). Area A to E are situated within the sampling trench previously milled for stable isotopes. Area F is on the surface of the sample. Analysis of all Raman spectra measured reveals calcite as the only mineral phase present (see S2 and S3 Figs). (TIF) [file pone.0273984.s001.tif]

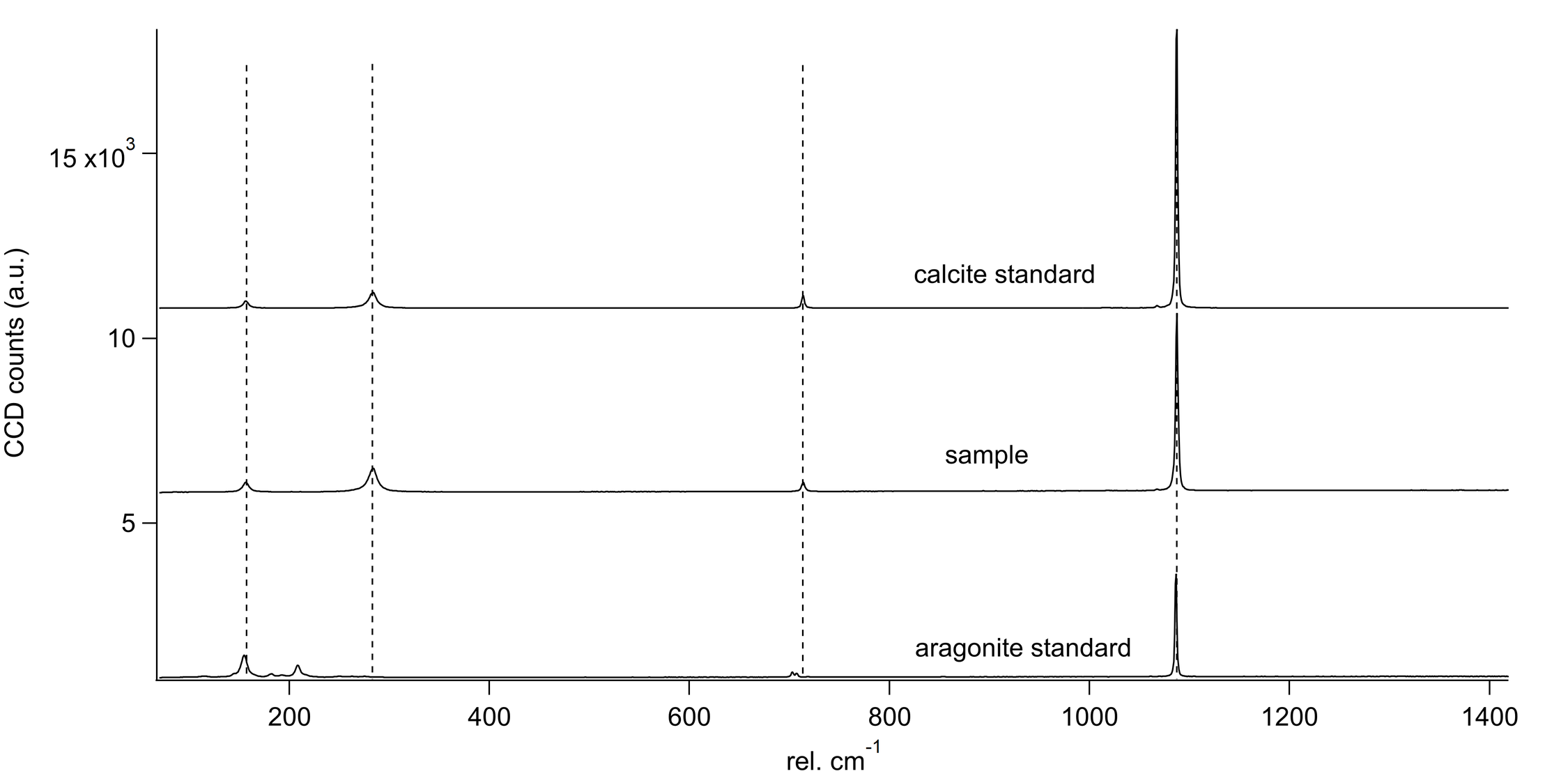

Supplement: S2 Fig — All areas have been identified as calcite and no traces of aragonite have been found. (TIF) [file pone.0273984.s002.tif]

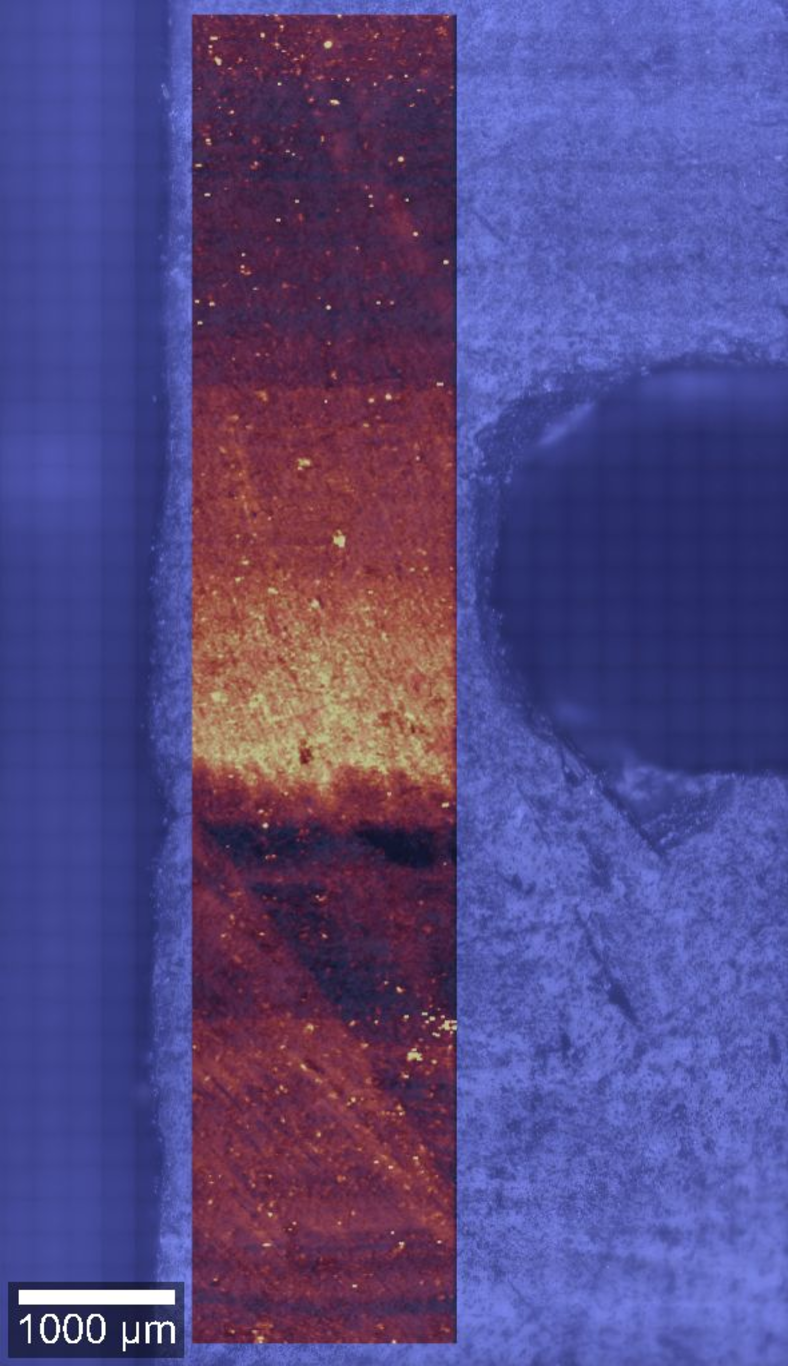

Supplement: S3 Fig — Enhanced fluorescence in Raman spectra of carbonates is often attributable to the presence of organic molecules [4]. This could indicate that the distribution of organic molecules is quantitatively or qualitatively different between darker and the brighter structures. (TIF) [file pone.0273984.s003.tif]

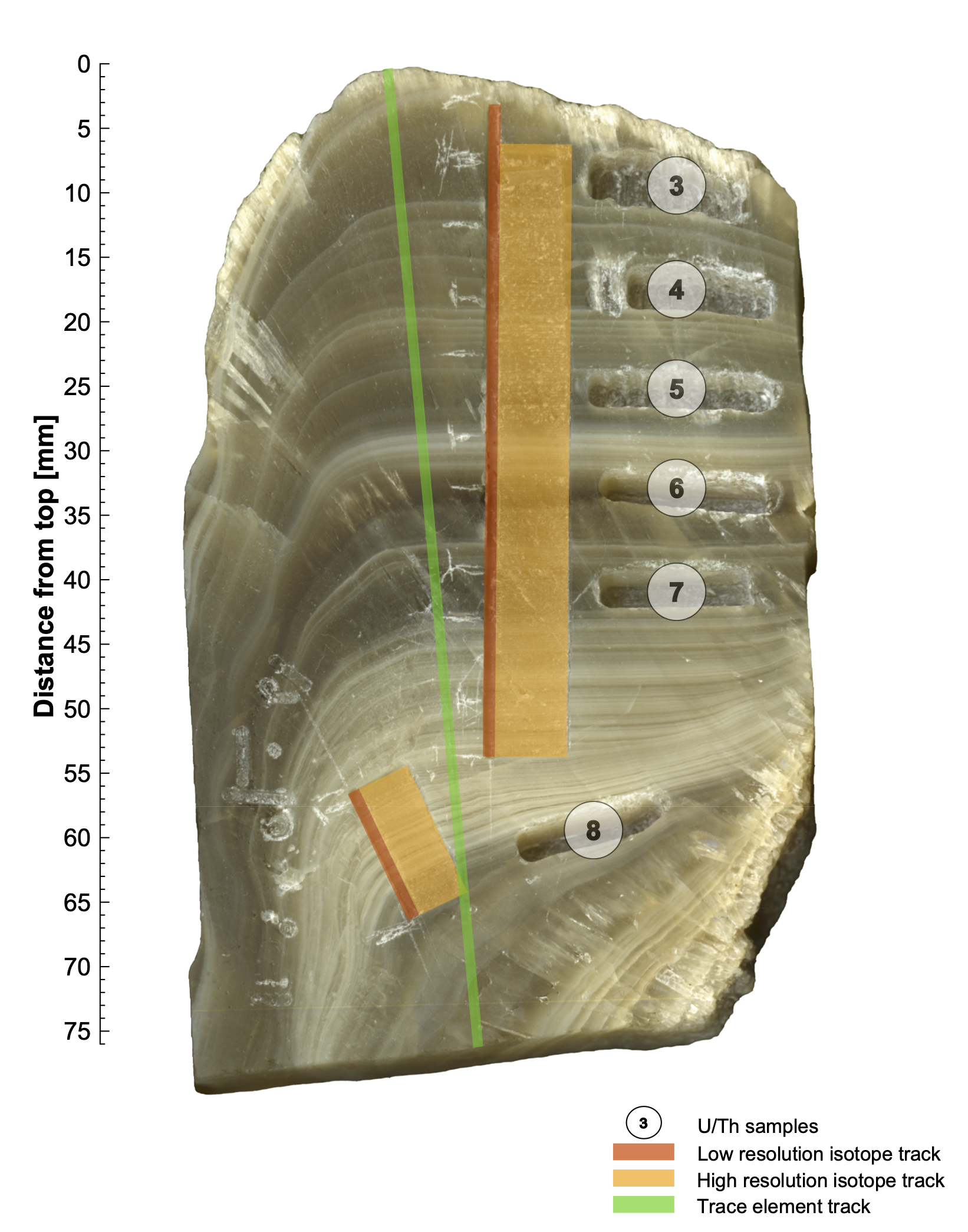

Supplement: S4 Fig — (TIF) [file pone.0273984.s004.tif]

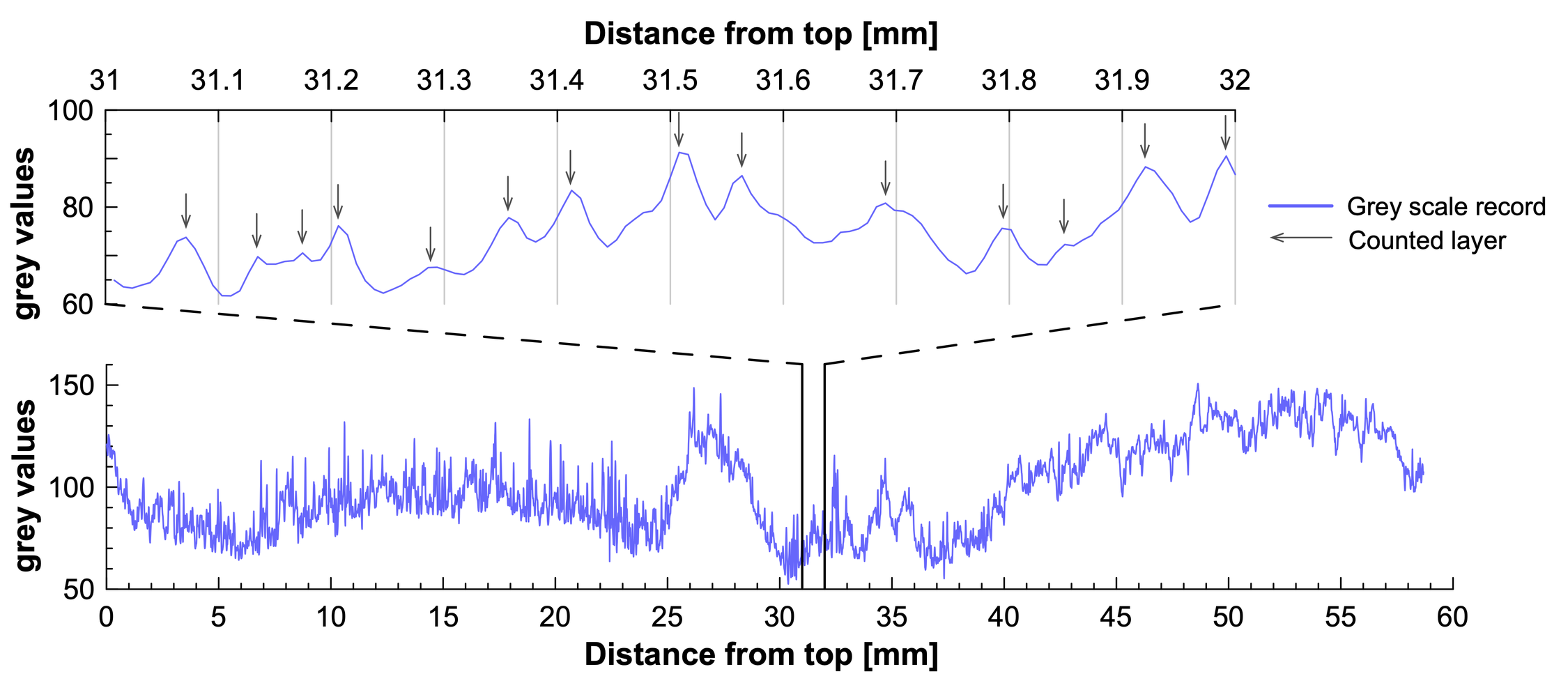

Supplement: S5 Fig — A magnified area provides an example of how layers were counted, with peaks labeled with black arrows. (TIF) [file pone.0273984.s005.tif]

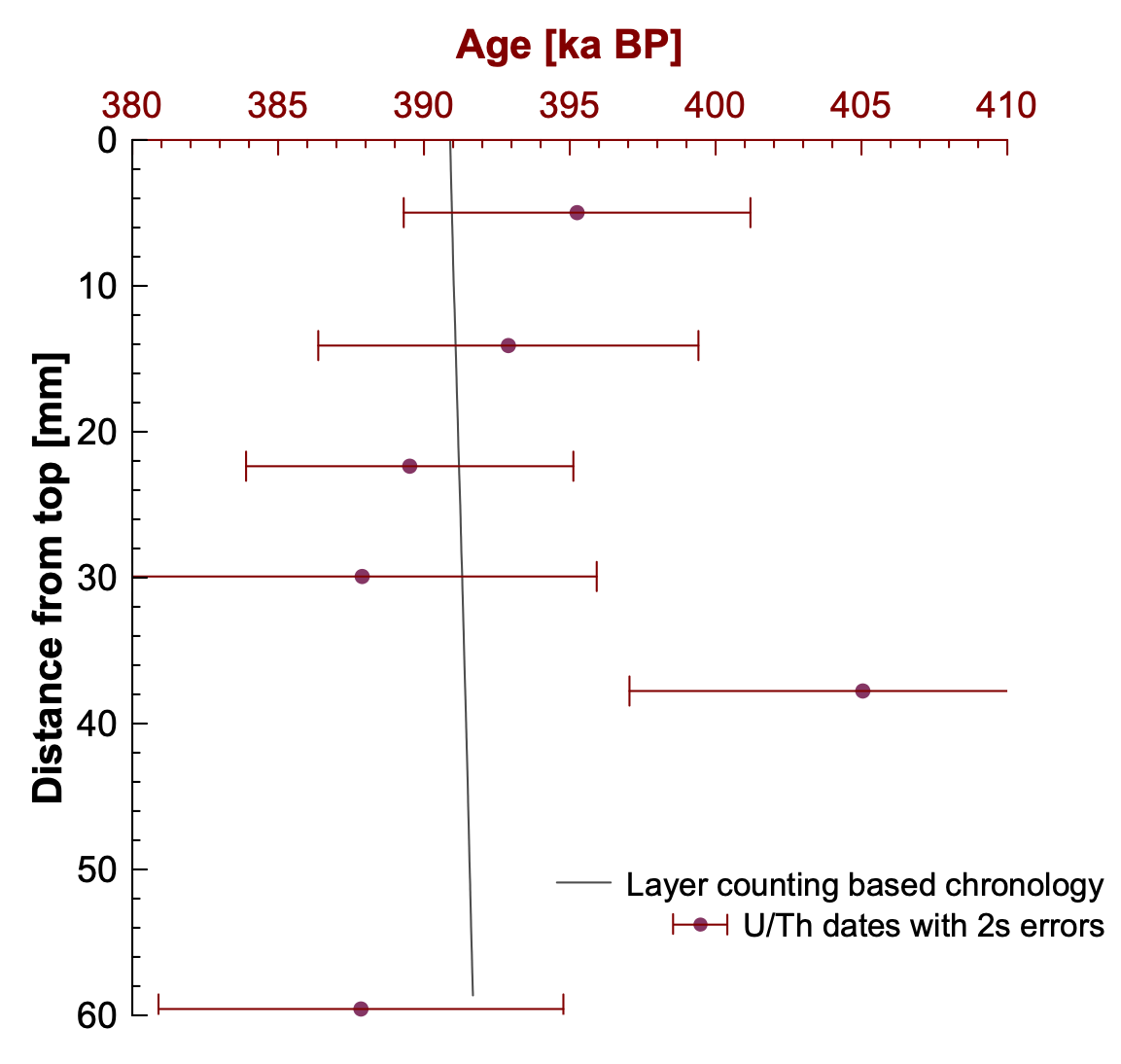

Supplement: S6 Fig — (TIF) [file pone.0273984.s006.tif]
