# Supplementary material for: Paleolithic occupation of arid Central Asia in the Middle Pleistocene
Source: PLoS One. 2022 Oct 21;17(10):e0273984. doi: 10.1371/journal.pone.0273984 (PMC9586385; doi:10.1371/journal.pone.0273984)
Supplement: S1 File — (DOCX) [file pone.0273984.s011.docx]

**S1 File. Confocal Raman microscopy methodology.**

Confocal Raman microscopy (CRM) allows to identify different mineral phases within a sample with high spatial resolution (few µm) [e.g., 1 and 2]. Raman imaging was performed using a WITec alpha R (WITec GmbH, Germany) confocal Raman microscope. All measurements are performed using a Zeiss Epiplan lens (20x, 0.4 NA), an excitation wavelength of 488 nm, and an ultra-high throughput spectrometer (UHTS 300, WITec, Germany) with a grating of 1800 g mm^-1^, 500 nm blaze.

The Raman spectra of calcite and aragonite show clear differences which allow to identify their presence at sub-µm to µm scale [3]. Calcite can be identified using the two lattice modes (translation mode T_c_, 155 cm^−1^ and librational mode L_c_, 282 cm^−1^) and the two internal modes (in-plane band ν_4_, 711 cm^−1^ and symmetric stretch ν_1_, 1085 cm^−1^). Aragonite was identified by the two lattice modes (translation mode T_a_, 152 cm^−1^ and librational mode L_a_, 206 cm^−1^) and the two internal modes (in-plane band ν_4_, 705 cm^−1^ and symmetric stretch ν_1_, 1085 cm^−1^).

We mapped six areas along the growth axis of stalagmite S-12-4 using CRM (figure S1, A to F).

**Supplemental references**

1. Nehrke G, Nouet J. Confocal Raman microscope mapping as a tool to describe different mineral and organic phases at high spatial resolution within marine biogenic carbonates: case study on Nerita undata (Gastropoda, Neritopsina). Biogeosciences. 2011;8(12):3761-9. doi: 10.5194/bg-8-3761-2011.

2. Milano S, Nehrke G. Microstructures in relation to temperature-induced aragonite-to-calcite transformation in the marine gastropod Phorcus turbinatus. PLOS ONE. 2018;13(10):e0204577. doi: 10.1371/journal.pone.0204577

3. Ramesh K, Melzner F, Griffith AW, Gobler CJ, Rouger C, Tasdemir D, et al. In vivo characterization of bivalve larval shells: a confocal Raman microscopy study. Journal of The Royal Society Interface. 2018;15(141).

4. Wall M, Nehrke G. Reconstructing skeletal fiber arrangement and growth mode in the coral Porites lutea (Cnidaria, Scleractinia): a confocal Raman microscopy study. Biogeosciences. 2012;9(11):4885-95. doi: 10.5194/bg-9-4885-2012.
